# Baseline Results Investigate APOE‐Memory Connection in Latin‐American Population at Risk for Dementia ‐ LatAm‐FINGERS Initial Insights

**DOI:** 10.1002/alz70858_107372

**Published:** 2025-12-25

**Authors:** Clarisse Vasconcelos Friedlaender, Leonardo Cruz de Souza, Maira Tonidandel Barbosa, Karina Braga Gomes, Jessica Abdo Goncalves Tosatti, João Victor de Faria Rocha, Davi Melillo Cardoso, Marcelo Machado Prates, Andre Luis Lopes, Vitoria Silva Vieira, Vinicius Ribeiro Jeunon, Letícia Maria Ferreira de Souza, Lais Soares Figueiredo, Andreia de Andrades, Dias Teixeira, Ana Luisa Sosa, Ricardo Nitrini, Ricardo Allegri, Gustavo Sevlever, David Fernando Aguillón Niño, Daisy M Acosta, Luc¡a Crivelli, Ana Charamelo, Ivonne Z. Jimenez‐Velazquez, Nilton Custodio, Lissette Duque, Jorge M Leon‐Salas, Carolina Delgado, María Isabel Cusicanqui, Belen Custodio, Rosa Maria Salinas‐Contreras, Gustavo Henrique Cançado, Ana Vigil‐Martinez, Maria Da Graça Morais Martin, Andrés Damian, Ezequiel Ignacio Surace, Natalia Acosta‐Baena, Ismael Luis Calandri, Monica Sanches Yassuda, Claudia Kimie Suemoto, Maria Eugenia Martin, Sonia Maria Dozzi Brucki, Heather M Snyder, Maria C. Carrillo, Miia Kivipelto, Paulo Caramelli

**Affiliations:** ^1^ Universidade Federal de Minas Gerais, Belo Horizonte, Minas Gerais, Brazil; ^2^ Federal University of Minas Gerais, Belo Horizonte, Minas Gerais, Brazil; ^3^ Faculty of Medicine ‐ Federal University of Minas Gerais, Belo Horizonte, Minas Gerais, Brazil; ^4^ Faculdade de Medicina, Universidade Federal de Minas Gerais, Belo Horizonte, Brazil, Belo Horizonte, Minas Gerais, Brazil; ^5^ School of Physical Education, Physiotherapy and Occupational Therapy ‐ Federal University of Minas Gerais, Belo Horizonte, Minas Gerais, Brazil; ^6^ Universidade Federal de Minas Gerais, Belo Horizonte, Minas gerais, Brazil; ^7^ Behavioral and Cognitive Neurology Unit, Faculdade de Medicina, Universidade Federal de Minas Gerais, Belo Horizonte, Brazil, Belo Horizonte, Minas Gerais, Brazil; ^8^ Instituto Nacional de Neurología y Neurocirugía, Mexico City, DF, Mexico; ^9^ Faculdade de Medicina da Universidade de São Paulo, São Paulo, Brazil; ^10^ Fleni, Buenos Aires, Argentina; ^11^ Fleni, CABA, Buenos Aires, Argentina; ^12^ Grupo Neuropsicología y Conducta, Facultad de Medicina, Universidad de Antioquia, Medellin, Colombia; ^13^ Universidad Nacional Pedro Henriquez Urena (UNPHU), Santo Domingo, Santo Domingo, Dominican Republic; ^14^ Departamento de Neuropsicología, Facultad de Medicina‐Hospital de Clínicas, Universidad de la República, Montevideo, Uruguay; ^15^ University of Puerto Rico, School of Medicine, San Juan, Puerto Rico; ^16^ Unidad de Investigación de Deterioro Cognitivo y Prevención de Demencia, Instituto Peruano de Neurociencias, Lima, Lima, Peru; ^17^ Neuromedicenter, Quito, Ecuador; ^18^ Clínica Bíblica Hospital, San José, Costa Rica; ^19^ Global Brain Health Institute, Trinity College Dublin, dublin, Ireland; ^20^ Hospital Clínico Universidad de Chile, Departamento de Neurología y Neurocirugía, Santiago, Santiago, Chile; ^21^ Centro Neurológico Mente Activa, La Paz, Bolivia (Plurinational State of); ^22^ Laboratorio de demencias del Instituto Nacional de Neurología y Neurocirugía Manuel Velasco Suárez, Ciudad de Mexico, Mexico; ^23^ Escola de Educação Física, Fisioterapia e Terapia Ocupacional, Universidade Federal de Minas Gerais, Belo Horizonte, Brazil, Belo Horizonte, Minas Gerais, Brazil; ^24^ Laboratorio de demencias del Instituto Nacional de Neurología y Neurocirugía Manuel Velasco Suárez, México, Mexico city, DF, Mexico; ^25^ LIM44, Hospital das Clinicas HCFMUSP, Faculdade de Medicina, Universidade de Sao Paulo, Sao Paulo, Sao Paulo, Brazil; ^26^ CUDIM Centro Uruguayo de Imagenología Molecular, Montevideo, Uruguay, Montevideo, Uruguay; ^27^ Fleni, Buenos Aires, Buenos Aires, Argentina; ^28^ Grupo de Neurociencias de Antioquia, Facultad de Medicina, Universidad de Antioquia, Medellín, Antioquia, Colombia; ^29^ Universidade de São Paulo, São Paulo, São Paulo, Brazil; ^30^ Division of Geriatrics, Department of Internal Medicine, University of São Paulo Medical School, São Paulo, São Paulo, Brazil; ^31^ FLENI, Buenos Aires, Argentina; ^32^ Cognitive and Behavioral Neurology Unit, Hospital das Clinicas HCFMUSP, Faculdade de Medicina, Universidade de Sao Paulo, Sao Paulo, Sao Paulo, Brazil; ^33^ Alzheimer's Association, Chicago, IL, USA; ^34^ Division of Clinical Geriatrics, Center for Alzheimer Research, Department of Neurobiology, Care Sciences and Society, Karolinska Institutet, Stockholm, Stockholm, Sweden

## Abstract

**Background:**

Latin America (LA) faces heightened vulnerability to modifiable dementia risk factors. Early identification of at‐risk individuals is crucial for implementing effective preventive strategies. The Free and Cued Selective Reminding Test with Immediate Recall (FCSRT‐IR) is promising in early detection of mnemonic impairment, with high sensitivity and specificity for Alzheimer's Disease (AD) risk. While sociodemographic and genetic factors, including APOE ε4 genotype, are associated with elevated dementia risk, their influence on cognitive performance in LA populations remains unclear. We aim to investigate sociodemographic factors and APOE ε4 genotype influence on mnemonic performance in high‐risk LA individuals.

**Method:**

Cross‐sectional analysis of LatAm‐FINGERS baseline data, a randomized, multicenter trial evaluating non‐pharmacological interventions for cognitive decline prevention in LA. Inclusion criteria: age 60‐77 years; CAIDE ≥ 6; ‐1.5 ≤ z score ≤ 0 on MMSE or CERAD word list. Exclusion criteria: MMSE < 20; dementia; illiteracy. Participants from Argentina, Brazil, Chile, and Uruguay were included. Memory assessment used FCSRT‐IR and SOMI (Stages of Objective Memory Impairment) classification. APOE genotyping was performed on blood samples using PCR‐RFLP analysis. Jamovi software (v2.3) analyzed correlations and associations (*p* < 0.05).

**Result:**

Sample (*N* = 358 participants): age 67.75 ± 4.86 years; education 13.41 ± 3.14 years; 72.3% female; 61.2% white; 22.3% APOE ε4 carriers. FCSRT‐IR scores correlated significantly with sociodemographics (*p* < .001) and varied among countries (*p* < .001). SOMI distribution: 58% SOMI‐0 (preserved memory), 27.6% SOMI‐1/2 (mild impairment). Argentina showed 36% in SOMI‐3/4 (severe impairment); Uruguay 75% in SOMI‐0. No significant sociodemographic differences were found between APOE ε4 carriers/non‐carriers. Non‐carriers performed better on FCSRT‐IR, significant only for identification score (*p* = .017). No significant APOE ε4‐SOMI association.

**Conclusion:**

Sociodemographic profile significantly influences memory performance, while APOE ε4 genotype shows limited association. This suggests environmental and social factors may play a more central role in cognitive performance than genetic predisposition in LA populations.

